# Preparation, characterization, and osteogenic activity mechanism of casein phosphopeptide-calcium chelate

**DOI:** 10.3389/fnut.2022.960228

**Published:** 2022-08-02

**Authors:** Wen Huang, Linhui Lao, Yuliang Deng, Ziwei Li, Wanwen Liao, Shan Duan, Suyao Xiao, Yong Cao, Jianyin Miao

**Affiliations:** ^1^Guangdong Provincial Key Laboratory of Nutraceuticals and Functional Foods, College of Food Science, South China Agricultural University, Guangzhou, China; ^2^State Key Laboratory of Natural Medicines, China Pharmaceutical University, Nanjing, China; ^3^State Key Laboratory for Chemistry and Molecular Engineering of Medicinal Resources (Guangxi Normal University), Guilin, China; ^4^Solid-State Fermentation Resource Utilization Key Laboratory of Sichuan Province, Yibin, China

**Keywords:** CPP-Ca, preparation, characterization, osteogenic activity, RNA-seq, active mechanism

## Abstract

Casein phosphopeptides (CPPs) are good at calcium-binding and intestinal calcium absorption, but there are few studies on the osteogenic activity of CPPs. In this study, the preparation of casein phosphopeptide calcium chelate (CPP-Ca) was optimized on the basis of previous studies, and its peptide-calcium chelating activity was characterized. Subsequently, the effects of CPP-Ca on the proliferation, differentiation, and mineralization of MC3T3-E1 cells were studied, and the differentiation mechanism of CPP-Ca on MC3T3-E1 cells was further elucidated by RNA sequencing (RNA-seq). The results showed that the calcium chelation rate of CPPs was 23.37%, and the calcium content of CPP-Ca reached 2.64 × 10^5^ mg/kg. The test results of Ultraviolet–Visible absorption spectroscopy (UV) and Fourier transform infrared spectroscopy (FTIR) indicated that carboxyl oxygen and amino nitrogen atoms of CPPs might be chelated with calcium during the chelation. Compared with the control group, the proliferation of MC3T3-E1 cells treated with 250 μg/mL of CPP-Ca increased by 21.65%, 26.43%, and 28.43% at 24, 48, and 72 h, respectively, and the alkaline phosphatase (ALP) activity and mineralized calcium nodules of MC3T3-E1 cells were notably increased by 55% and 72%. RNA-seq results showed that 321 differentially expressed genes (DEGs) were found in MC3T3-E1 cells treated with CPP-Ca, including 121 upregulated and 200 downregulated genes. Gene ontology (GO) revealed that the DEGs mainly played important roles in the regulation of cellular components. The enrichment of the Kyoto Encyclopedia of Genes and Genomes Database (KEGG) pathway indicated that the AMPK, PI3K-Akt, MAPK, and Wnt signaling pathways were involved in the differentiation of MC3T3-E1 cells. The results of a quantitative real-time PCR (qRT-PCR) showed that compared with the blank control group, the mRNA expressions of Apolipoprotein D (APOD), Osteoglycin (OGN), and Insulin-like growth factor (IGF1) were significantly increased by 2.6, 2.0 and 3.0 times, respectively, while the mRNA levels of NOTUM, WIF1, and LRP4 notably decreased to 2.3, 2.1, and 4.2 times, respectively, which were consistent both in GO functional and KEGG enrichment pathway analysis. This study provided a theoretical basis for CPP-Ca as a nutritional additive in the treatment and prevention of osteoporosis.

## Introduction

Osteoporosis is a disease characterized by the loss of bone mass and the increase of bone fragility (1). In a normal state, bone tissues are in a dynamic equilibrium maintained by a combination of bone formation and bone resorption (2), during which osteoclasts absorb old bone to form lacunae and osteoblasts form new bone in lacunae to fill the lacunae (3–6). The dynamic imbalance between osteoblasts and osteoclasts is one of the main causes of osteoporosis. Therefore, promoting the proliferation and differentiation of osteoblasts is conducive to the prevention and treatment of osteoporosis (7). At present, the treatments of osteoporosis are mainly drug therapy, such as bisphosphonates, estrogen, calcitonin, and fluoride (8). However, long-term use of these drugs might bring side effects, such as sciatica, increased risk of breast cancer, intestinal diseases, and so on (9, 10). Therefore, it is of great practical significance to find appropriate methods to prevent or treat osteoporosis. Studies have shown that the natural active peptide calcium chelate has the advantages of improving the bioavailability of calcium, promoting the proliferation and differentiation of osteoblasts, and having a preventive and therapeutic effect on osteoporosis. For example, egg white peptide-calcium chelate can promote the ALP activity in HFOB cells (11), and porcine bone collagen peptide calcium chelate promotes osteoblast proliferation and differentiation by activating the PI3K/Akt signaling pathway (12).

Casein, the main protein in milk, can be hydrolyzed by proteases to produce bioactive peptides, of which CPPs have been extensively studied due to their good solubility, digestive stability, and strong ability to promote calcium absorption (13). For example, Liu et al. found that CPPs can promote the transport of calcium in Caco-2 monolayers (14). Liao et al. purified and identified a novel calcium-binding peptide with good calcium transport capacity from casein hydrolysate (15). Studies have shown that CPPs have good osteogenic activity. Liu et al. found that femoral bone index, serum calcium, serum osteocalcin levels, and femoral calcium content were significantly increased in rats after 7 weeks of high-dose CPPs feeding (16). Pan et al. found a significant proliferative effect of casein hydrolysate on osteoblasts (HFOB1.19) (17). It was also found that because these phosphorylated peptides are negatively charged, they can chelate with some minerals, such as iron and calcium, and have a strong binding capacity, greatly increasing the solubility and bioavailability of minerals (13). However, current studies on CPP-Ca mainly focus on the ability to promote calcium absorption. Li et al. used the Caco-2 monolayer cell model to find that CPP-Ca was superior to calcium chloride, L-aspartate calcium, and CPPs mixed with calcium chloride in promoting calcium absorption (18). CPP-Ca has been less studied on osteoblasts (MC3T3-E1), while the mechanism of osteogenic activity of CPP-Ca has not been clarified.

RNA-seq is a deep sequencing mode that can be used to evaluate a complete set of organisms' transcriptional genes or transcriptome and non-coding RNA. With high sensitivity, high repeatability, high throughput, and affordability, RNA-seq has become a standard technology for genomics transcriptome analysis (19–21). Bone formation is a dynamic and complex process, and RNA-seq can help us to deepen our understanding of the mechanism of CPP-Ca-induced osteogenic activity of MC3T3-E1 cells.

Wu et al. found that phosphorylation of peptides significantly improved their calcium-peptide binding capacity and osteogenic activity (22). The CPPs are an excellent choice for the preparation of calcium peptide chelate due to their natural property of the phosphate group. In addition, our team has been researching the activity of CPPs, and we are committed to developing a product related to casein phosphopeptide-calcium chelate with osteogenic activity. In this study, the preparation of CPP-Ca was optimized, and the chelating properties of CPP-Ca were characterized. Moreover, the effects of CPP-Ca on proliferation, differentiation, and mineralization of MC3T3-E1 cells were detected, and RNA-seq technology was used to reveal the differentiation mechanism of CPP-Ca on MC3T3-E1 cells. This study provided a theoretical basis for CPP-Ca as a functional food in treating and preventing osteoporosis.

## Materials and methods

### Materials and reagents

Casein and trypsin (10,000 U/g) were provided by Green Extraction Biotechnology Co., Ltd. Alpha modification of Eagle's minimum essential medium (α-MEM) and fetal bovine serum (FBS) were purchased from GIBCO (Grand Island, USA). β-glycerophosphate, L-ascorbic acid, and methylthiazolyldiphenyl-tetrazolium bromide (MTT) were purchased from Macklin (Shanghai, China). Paraformaldehyde and alizarin red (0.1%, pH 4.2) were purchased from Yuanye (Shanghai, China). All other chemicals were of the highest grade available commercially.

### Preparation of casein phosphopeptides (CPPs)

The casein was mixed with the trypsin (1.0%) and digested under certain conditions (substrate concentration 10%, pH 8.0, temperature 50°C) for 3 h. Following hydrolysis, the mixture was heated at 90°C for 10 min to inactivate the enzyme and cooled to room temperature. After adjusting pH to 4.6, the hydrolysate was centrifuged at 4,000 r/min for 10 min, and the supernatant was lyophilized to obtain CPPs.

### Preparation of CPP-Ca

#### Single-factor and orthogonal experiment

Lyophilized CPPs were dissolved in deionized water, then calcium chloride was added, and the mass ratio of peptide:calcium was 2:1. The solution was adjusted to different pH values (4–8), and stirred at different temperatures (35–55°C) for different times (50–130 min). Subsequently, absolute ethanol (5 times the volume of the solution) was added to separate the peptide calcium chelate from free calcium ions. Then, the mixture was centrifuged at 4,000 r/min for 20 min, the supernatant was discarded, and the dried precipitation was CPP-Ca.

Combined with the results of the single-factor experiments, the optimal conditions for the preparation of CPP-Ca were determined by an orthogonal experiment (3-factor 3-level). In this design, pH (A), chelation temperature (B), and time (C) were chosen as independent variables, and chelation rate was chosen as the evaluation index.

#### Determination of calcium chelation activity

Calcium chelating activity was determined by the o-cresol phthalein colorimetry method (15). Five milligrams of CPPs freeze-dried powder were mixed with 1 mL of 5 mM CaCl_2_ solution and 2 mL of phosphate buffer. The mixture was shaken at 37°C for 60 min. The solution was then centrifuged at 4,000 r/min for 20 min; the supernatant was diluted and mixed with the working solution. After reaction for 2 min, the absorbance value was measured at 570 nm using a microplate reader (Beaconsfield, U.K.).


$$\begin{array}{r} Calcium\;chelation\;rate\;\left(\%\right)=\frac{M1}{M2}\times 100\% \end{array}$$

M1: calcium content in the sample, μg.

M2: calcium content added to the system, μg.

#### Determination of calcium content

Calcium content was determined by the flame atomic absorption spectrometry (23). The lyophilized CPP-Ca was dissolved in deionized water, and the content of calcium was assayed using the atomic absorption spectrophotometer AA- 6300C (Shimadzu, Japan) after mixed-acid digestion (HNO_3_:HCl, 1:3, v/v).

### Characterization of CPP-Ca

#### Ultraviolet absorption spectrum analysis

UV absorption spectra of CPPs and CPP-Ca were determined by UV spectrophotometer UV1750 (Shimadzu, Japan). 1 mg/mL CPPs and CPP-Ca solutions were prepared, and scanning was performed in the spectral region of 200–400 nm.

#### Fourier transform infrared spectroscopy analysis

Two milligrams of lyophilized CPPs and CPP-Ca were, respectively, mixed with 100 mg of dried KBr. All FTIR spectra were measured by an FTIR spectrometer Vertex 70 (Brook, Germany) within a scope of 4,000 cm^−1^ to 400 cm^−1^.

#### Cells culture and MTT assay

The mouse pre-osteoblast cell line MC3T3-E1 subclone in 14 cells (Cell Bank, Shanghai Institutes for Biological Sciences, Shanghai, China) were cultured in α-MEM supplemented with 1% antibiotic–antimycotic solution and 10% FBS in an atmosphere of 5% CO_2_ at 37°C. When cells reached 80–90% confluence, they were sub-cultured by treatment with 0.25% trypsin-EDTA and grown in sterile tissue culture plates.

The MTT assay was according to a previous study by Liao et al. (24). Specifically, MC3T3-E1 cells were seeded in a 96-well plate at a density of 5 × 10^3^ cells/well. After 24 h of incubation, the culture medium was discarded and replaced by the medium containing different concentrations of CPP-Ca (0, 50, 100, 150, 200, and 250 μg/mL) to incubate for 24, 48, and 72 h. After incubation, 100 μL MTT (0.5 mg/mL) was added for 4 h at 37°C. The supernatant was removed, 150 μL of dimethyl sulfoxide was added to each well and shaken on an oscillator for 10 min. The optical density was recorded at the wavelength of 570 nm by a microplate reader (Beaconsfield, U.K.). Six parallel experiments were tested for each cultivating period.

#### Determination of ALP activity

The ALP activity assay was performed according to a previous study (25). MC3T3-E1 cells were seeded in a 6-well plate at a density of 1 × 10^6^ cells/well to cultivate for 24 h. After incubation, the culture medium was discarded and replaced by the differentiation medium (α-MEM containing 10% FBS, 1% antibiotic-antimycotic solution, 10 mM sodium β-glycerol phosphate, and 50 μg/mL L-ascorbic acid) containing different concentrations of CPP-Ca (0, 50, 100, 150, 200, and 250 μg/mL). The medium was replaced every other day for 7 days. After 7 days, protein concentrations and ALP activity were determined using the BCA Protein Assay Kit and the Alkaline Phosphatase Assay Kit (Jiancheng Biological Technology, Nanjing, China), respectively.

#### Alizarin red staining and quantification

The mineralization studies were performed according to a previous study (25). MC3T3-E1 cells were seeded in 6-well plate at a density of 1 × 10^6^ cells/well. After 24 h of incubation, the culture medium was discarded and replaced by the differentiation medium containing different concentrations of CPP-Ca (0, 100, and 250 μg/mL). The medium was replaced every other day for 21 days. After 21 days, the cells were washed twice with phosphate-buffered saline (PBS), fixed with 4% paraformaldehyde for 30 min at 37°C, and rinsed with deionized water twice. One milliliter of alizarin red was added to each well, the cells were left to stain for 5 min, and then washed twice with deionized water. A phase-contrast microscope with a digital camera (Canon DS126201, Japan) was used to locate the calcium nodules, which were magnified (4X) and photographed. To quantify the alizarin red staining area, the calcium nodules were dissolved with 10% (w/v) cetylpyridinium chloride for 30 min, and the absorbance at 562 nm of the solubilized alizarin red was measured using a microplate reader (Beaconsfield, U.K.).

#### RNA-seq and data analysis

MC3T3-E1 cells were seeded in 6-well plate at a density of 1 × 10^6^ cells/well. After 24 h of incubation, the culture medium was discarded, and replaced by the differentiation medium containing 250 μg/mL CPP-Ca. After 7 days, total RNA was extracted. RNA purity and quantification were evaluated using the NanoDrop 2000 spectrophotometer (Thermo Scientific, USA). RNA integrity was assessed using the Agilent 2100 Bioanalyzer (Agilent Technologies, Santa Clara, CA, USA). Then, the libraries were constructed using the TruSeq Stranded mRNA LT Sample Prep Kit (Illumina, San Diego, CA, USA) according to the manufacturer's instructions. The transcriptome sequencing and analysis were conducted by the OE Biotech Co., Ltd. (Shanghai, China).

The libraries were sequenced on an Illumina HiSeq X Ten platform and 150 bp paired-end reads were generated. About 50 million reads, raw reads for each sample were generated. Raw data (raw reads) of fast q format were first processed using Trimmomatic (a flexible trimmer for Illumina sequence data), and the low-quality reads were removed to obtain the clean reads. Then, about 49 million clean reads for each sample were retained for subsequent analyses. The clean reads were mapped to the human genome (GRCh38) using HISAT2 (26). FPKM (27) of each gene was calculated using Cufflinks (28), and the read counts of each gene were obtained by HTSeqcount (29). Differential expression analysis was performed using the DESeq (2012) R package (30). *P* < 0.05 and fold change > 2 were set as the threshold for significantly differential expression. Hierarchical cluster analysis of DEGs was performed to demonstrate the expression pattern of genes in different groups and samples. GO enrichment and KEGG (31) pathway enrichment analysis of DEGs were performed, respectively, using R based on the hypergeometric distribution.

#### Quantitative real-time polymerase chain reaction (qRT-PCR)

Briefly, total RNA was extracted according to the manufacturer's protocol. And then, mRNA was reverse-transcribed into cDNA by Reverse Transcription Kit (TransGen Biotech, China) according to the instructions of the manufacturer. The primer sequence of the APOD, OGN, IGF1, LRP4, NOTUM, WIF1, and housekeeping gene β-actin are shown in Table 1. PCR reaction was carried out according to the method provided by Real-Time PCR Kit (TransGen Biotech, China). The normalized expression of validated genes was calculated based on the 2^−ΔΔCT^ method.

**Table 1:** Primers used for qRT-PCR for DEGs.

**Target gene**	**Sequences**	**Product**
		**length (bp)**
APOD	GACAACAACAGATCAAGCGA	92
	GGGAACGCAAAGCAAAGA	
IGF1	AGTCCATTAAGACGCACTTAC	93
	AAGAAACCAGGACTCCCAA	
OGN	AAAGTCTACGTGTAATTCACCT	82
	CGAGTGTCATTAGCCTTGC	
LRP4	CGCTGCTACTGAACAACC	81
	TGACATCCGACCAGAAGAC	
NOTUM	GGTGGAATGCCAATATGGT	83
	TTCATTCTTGTCAGACTTGGGT	
WIF1	GCAGGCAGAATACTTCTACGA	83
	AAGGGACATTGACAGTTGG	
β-actin	GGCTCCTAGCACCATGAAGA	187
	AGCTCAGTAACAGTCCGCC	

#### Statistical analysis

All experiments were conducted with three replicates (*n* = 3). The results were subjected to a one-way analysis of variance (ANOVA). A significant value of *p* < 0.05 was set for all statistical analysis.

## Results and discussion

### Optimization of CPP-Ca preparation process

#### Single-factor experimental analysis

**Figure 1:**
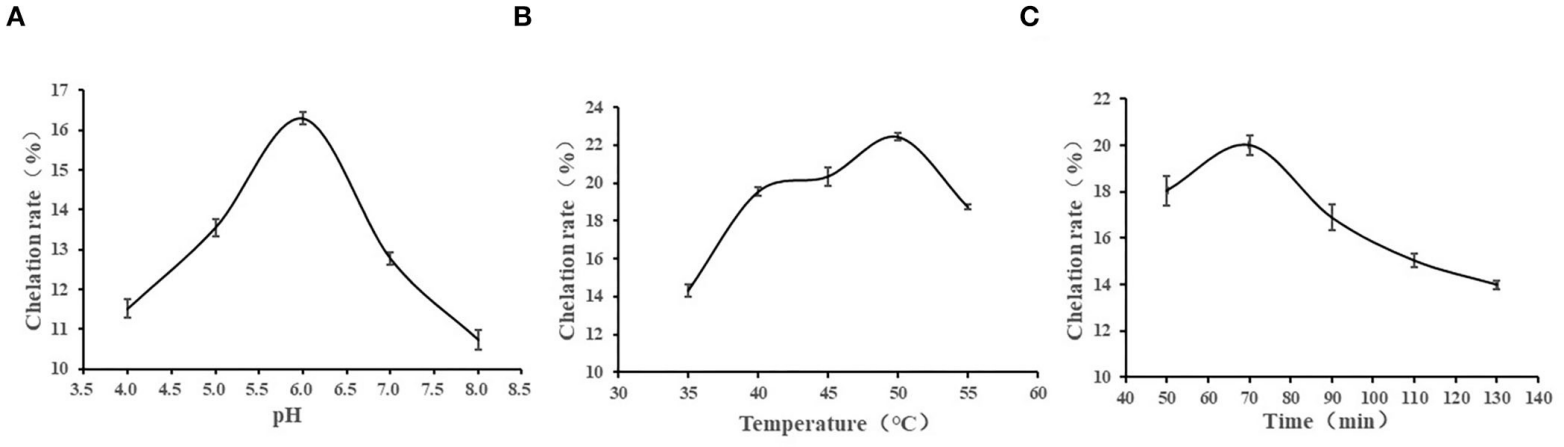
Effects of pH **(A)**, temperature **(B)**, and time **(C)** on chelation rate.

Figure 1 shows the effects of different chelating pH, temperature, and time on the calcium chelating rate of CPPs. The chelation rate increased as the pH raised, and the maximum value was obtained at pH 6 with a chelation rate of 11.52%. Then, it showed a downward trend (Figure 1A). In an acidic solution, there is a large amount of H^+^, which will compete with Ca^2+^, leading to a lower carboxyl coordination capacity of CPPs; therefore, the chelating capacity of CPPs in an acidic environment is weak. As the acidity diminished, the interaction between negatively charged COOH^−^ and Ca^2+^ was enhanced, so the highest chelation rate occurred at pH 6.0. In an alkaline environment, due to the presence of a large amount of OH^−^, they can react with Ca^2+^ to produce Ca(OH)_2_ precipitation, resulting in a gradual decline of the chelation rate (11). As can be seen from Figure 1B, the calcium chelation rate of CPPs increased as the temperature increased and reached a maximum value at 50°C and then decreased. These findings indicated that an appropriate temperature can promote molecular movement, which is conducive to the formation of chelate, but a high temperature will destroy the structure of the peptide and is not conducive to the chelation reaction (11). As shown in Figure 1C, when the reaction time was 70 min, the calcium chelation rate reached a maximum value, and excessive or too short a reaction time is not conducive to the generation of chelate. Excessive reaction time will destroy the stability of the reaction system, enhance the side reaction to a certain extent, and lead to the reduction of products (32).

#### Orthogonal experimental analysis

On the basis of single-factor experiments, orthogonal experiments were carried out at different temperature levels (47, 50, and 53°C), pH levels (5.5, 6.0, and 6.5), and chelating time levels (60, 70, and 80 min). The results of the orthogonal experiment are listed in Table 2. It can be seen that the influence of each factor on calcium chelation rate was in the following order: B > A > C according to the *R*-value, and the optimal level combination is A_2_B_3_C_1_. Under these conditions, the calcium chelation rate reached a maximum of 23.37%, and the calcium content was 2.64 × 10^5^ mg/kg.

**Table 2:** The analysis of orthogonal experiment results.

**No.**	**Influence factors**			**Calcium** **chelation** **rate/%**
	**Temperature** **(A)**	**pH** **(B)**	**Time** **(C)**	
1	1	1	1	11.33
2	1	2	2	15.84
3	1	3	3	19.32
4	2	1	2	14.46
5	2	2	3	14.57
6	2	3	1	23.83
7	3	1	3	5.89
8	3	2	1	13.99
9	3	3	2	14.57
K_1_	46.49	31.68	49.15	
K_2_	52.86	44.40	44.87	
K_3_	34.45	57.72	39.78	
R	18.41	26.04	9.37	
Primary and secondary order			B>A>C	
Theoretical optimal combination			A_2_B_3_C_1_	

*R refers to the result of extreme analysis*.

### Characterization of CPP-Ca

#### UV absorption spectrum analysis

**Figure 2:**
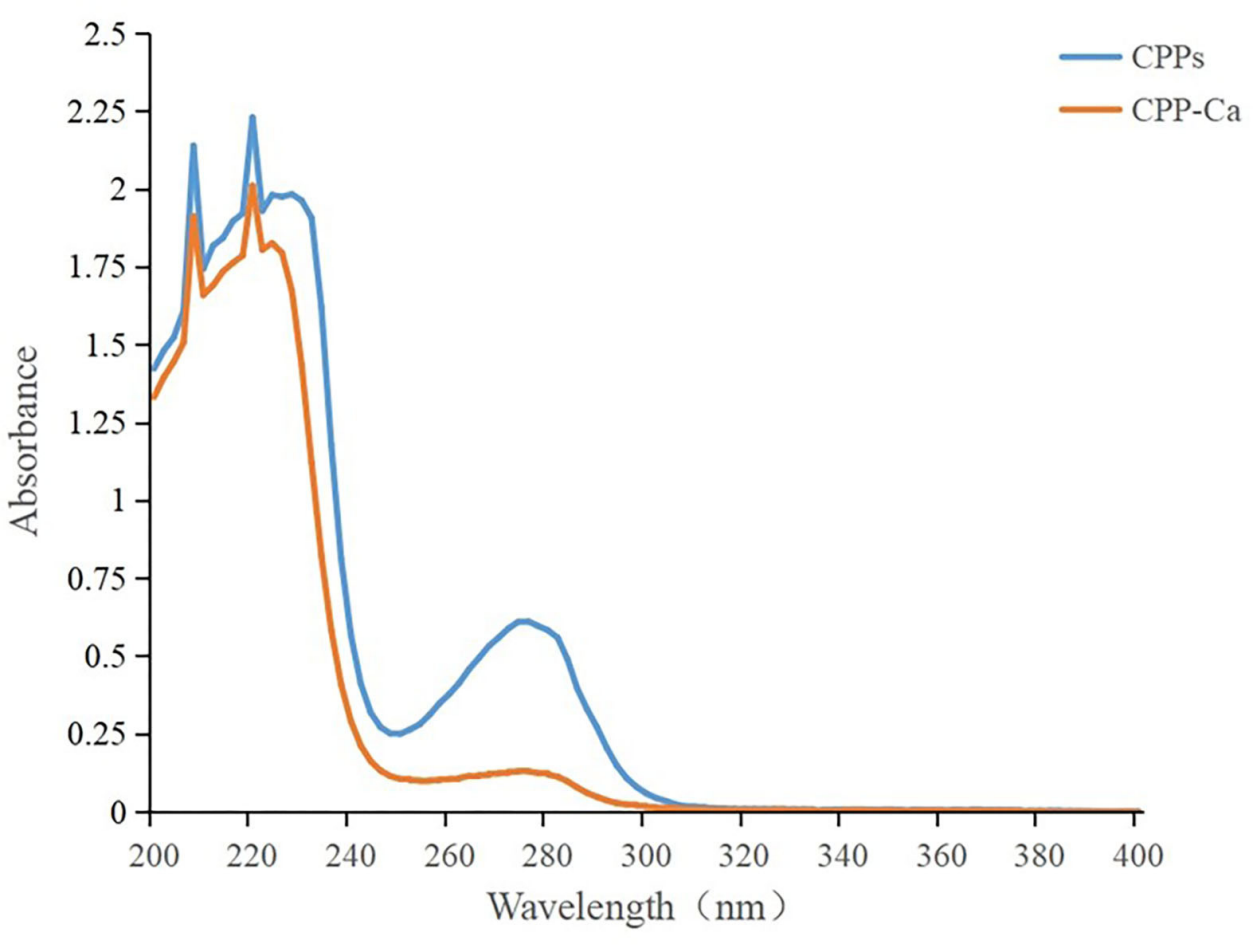
UV–Vis spectra of CPPs and CPP-Ca.

In ultraviolet spectrum analysis, the formation of the complex between organic ligand and metal ions will result in the transfer or disappearance of the original absorption peak or the emergence of a new absorption peak, which is often used in the identification and structural analysis of substances (33). As shown in Figure 2, the maximum absorption peaks of CPPs and CPP-Ca in the UV spectrum are at 220 nm, which corresponded to the characteristic peak of the peptide chain resulting from the n → π^*^ transition of C = O in the amide bonds (23). Compared with CPP-Ca, CPPs have a stronger absorption peak at about 280 nm, which is considered to be the characteristic peak of aromatic amino acids. The intensity of the absorption peak decreased significantly after chelation with calcium. A possible explanation was calcium ions combined with some aromatic amino acids on CPPs to form new chemicals, which affected the π → π^*^ electron transition of the conjugated double bond (34). Meanwhile, the spatial structure of CPPs changed with the chirality of Chromophores (C = O and -COOH) and Autochromes (-OH and -NH_2_) (35). In general, Ca^2+^ interacted with some aromatic amino acids on CPPs to form new chemicals, which involved C=O in amide bonds and led to the changes in the spatial structure of CPPs.

#### Fourier transform spectral analysis

**Figure 3:**
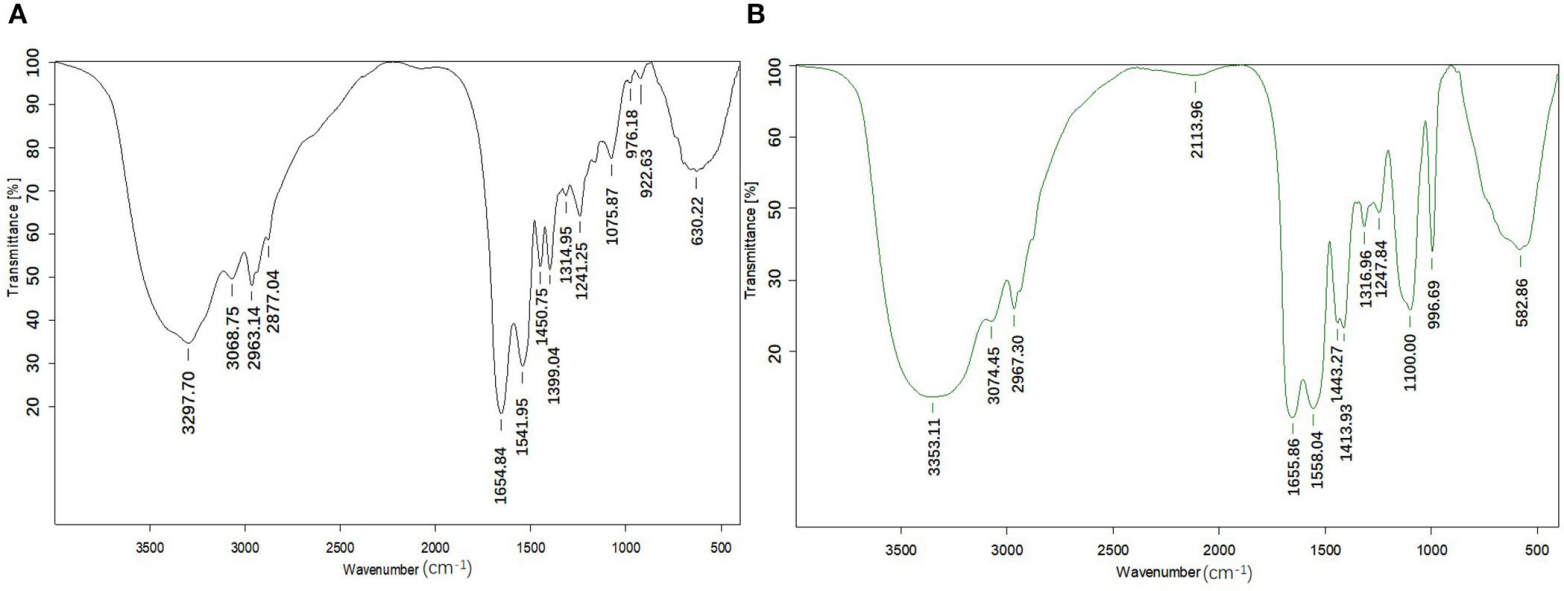
Infrared absorption spectra of CPPs and CPP-Ca (**(A)** CPPs; **(B)** CPP-Ca).

The position of wavenumber and the number and intensity of wave peak of infrared absorption band reflect the characteristics of molecular structure, so they are often used to identify the structural composition of substances or determine chemical groups. The differences in FTIR spectra between CPPs and CPP-Ca are displayed in Figure 3. CPPs have a significantly high-frequency absorption peak at 3,297.70 cm^−1^, which may be caused by the stretching vibration of N-H in CPPs. After chelating with calcium, the peak shifted to 3,353.11 cm^−1^, indicating that calcium ions reacted with N-H in CPPs, which is consistent with the phenomenon observed in pig collagen peptide calcium chelate (36). It has been reported that the formation of amide I bands is mainly due to stretching vibration of C=O, and includes the following secondary structures: β-turn, 1,700–1,660 cm^−1^; α-helix, 1,659–1,645 cm^−1^; irregular structure 1,644−1,640 cm^−1^; and β-sheet or extended structure 1,639−1,620 cm^−1^(37, 38). The amide I band of CPPs was detected around wave number 1,654.84 cm^−1^, while the amide I band of CPP-Ca appeared at 1,655.86 cm^−1^, suggesting the formation of peptide–calcium chelate was associated with C = O, and the complex have an α-helix structure. The vibration spectral region at 1,430–1,370 cm^−1^ is mainly caused by the stretching vibration of -COO- (39). After the interaction with calcium ions, the FTIR spectra shifted from 1,399.64 cm^−1^ to 1,413.93 cm^−1^, which may be due to the interaction between the positive divalent calcium ions and -COO- to form -COO- Ca. It has been reported that 1,060–1,100 cm^−1^ belongs to the O = P-O stretching region, which is associated with phosphate groups covalently bound to casein (40). The absorption peaks at 1,075.87 cm^−1^ and 1,100.00 cm^−1^ for CPPs and CPP-Ca, respectively, indicated that the amino acid residues in both CPPs and CPP-Ca are covalently bound to the phosphate group, and CPP-Ca retained the functional activity associated with the phosphate group. Previous research suggested the Ca–O vibrational band was between 500 and 800 cm^−1^. Meanwhile, the binding of the peptides with calcium ions may broaden and weaken of the peak, even the disappearance of some absorption peak (41). Through the difference analysis of the infrared spectrum, we found that the absorption peak of CPPs disappeared at 933.63 cm^−1^, and the original absorption peak (630.22 cm^−1^) shifted to 582.86 cm^−1^. Considering the reaction of the amide I band, it is speculated that this is due to the interaction between calcium ions and oxygen atoms in the carbonyl group. In general, the production of peptide calcium chelate involves the N-H, C=O, and -COO- groups, and the calcium ions interact with carboxyl oxygen and amino nitrogen atoms of CPPs.

### Effects of CPP-Ca on the proliferation of MC3T3-E1 cells

**Figure 4:**
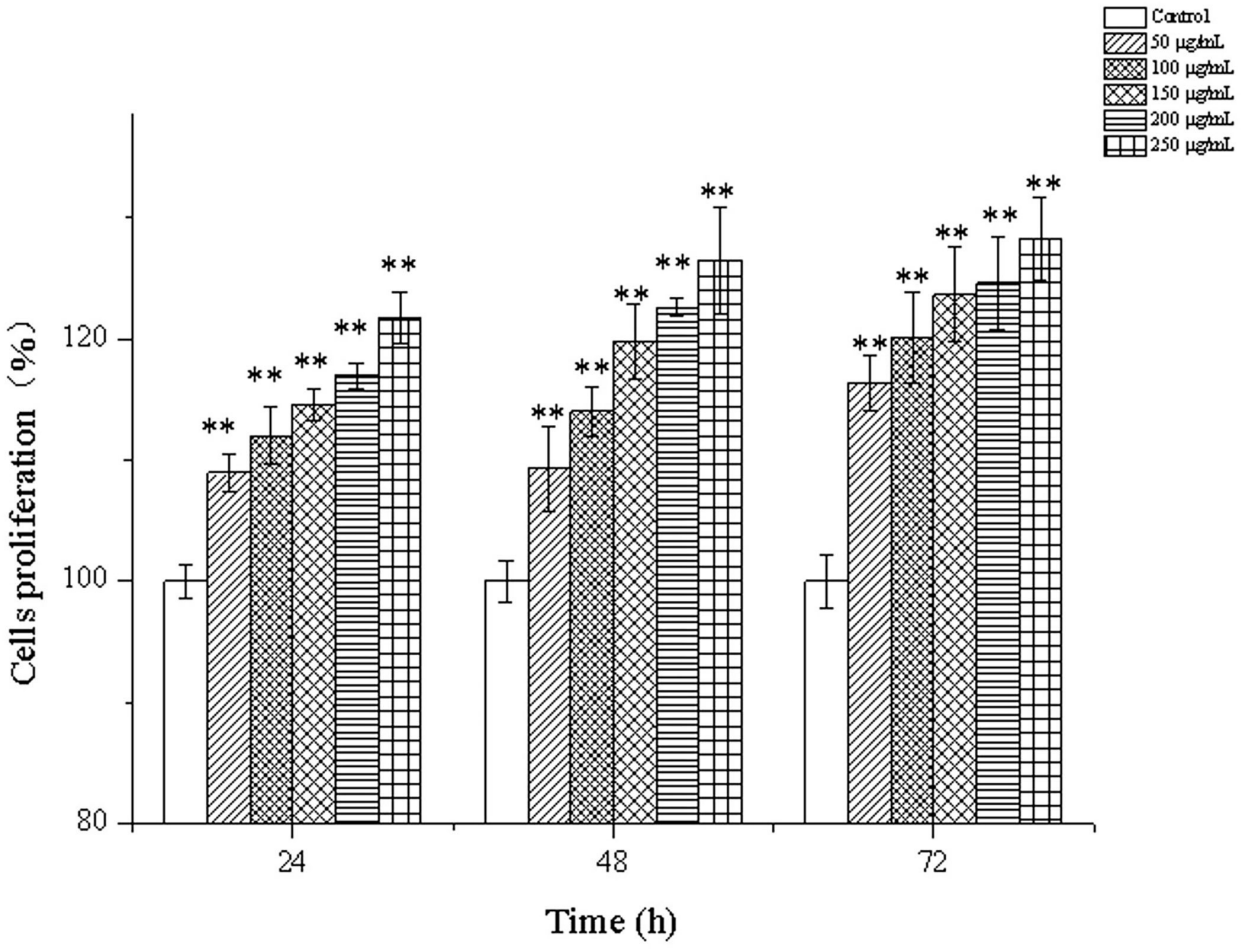
Cells proliferation of MC3T3-E1 cells treated with CPP-Ca (50, 100, 150, 200, and 250 μg/mL) at 24, 48, and 72 h. * *p* < 0.05; ** *p* < 0.01, compared with the control group.

The effects of CPP-Ca on the proliferation of MC3T3-E1 cells are shown in Figure 4. Compared with the blank control group, the proliferation of MC3T3-E1 cells increased with the increase of the treated concentrations of CPP-Ca and the treated time. The proliferation of MC3T3-E1 cells treated with 250 μg/mL CPP-Ca increased by 21.65, 26.43, and 28.43% at 24, 48, and 72 h, respectively. On the whole, CPP-Ca had positive effects on the proliferation of MC3T3-E1 cells. Wu et al. also found that porcine bone collagen peptide calcium chelate had a similar trend in promoting osteoblast proliferation, and the optimal concentration was 0.5 mg/mL, when the proliferation rate was around 140%. However, CPP-Ca achieved a similar effect at lower concentrations (22). Calcium phosphates have high solubility in water, such as a-tricalcium phosphate and tetracalcium phosphate. However, Atsushi et al. found that there was no significant difference in the cell number of MC3T3-E1 after 12 h, 1, 3, or 7 days treatment (42). When Liu et al. treated MC3T3-E1 cells with different concentrations of calcium chloride (1, 2, 4, and 8 mM), they found that the proliferation rate of MC3T3-E1 cells treated with 2 mM CaCl_2_ for 48 h and 72 h increased significantly reaching 113.1% and 124.4% (43). But the proliferation rate of MC3T3-E1 cells treated with 200 μg/ mL CPP-Ca for 48 h and 72 h reached more than 120%. Comparatively, CPP-Ca showed a strong ability to promote the proliferation of MC3T3-E1 cells.

### Effects of CPP-Ca on differentiation of MC3T3-E1 cells

**Figure 5:**
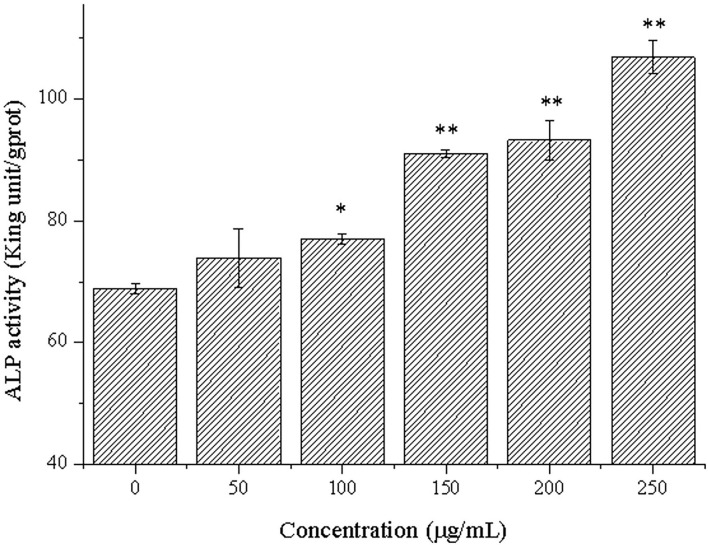
ALP activity of MC3T3-E1 cells treated with CPP-Ca (50, 100, 150, 200, and 250 μg/mL). *p < 0.05; **p < 0.01, compared with the control group.

ALP is a marker of early differentiation of osteoblasts (44). In addition, the higher the ALP activity was conducive to bone formation and repair (45). The effects of different concentrations of CPP-Ca on ALP activity of MC3T3-E1 cells are shown in Figure 5. It was noteworthy that CPP-Ca significantly improved the ALP activity of MC3T3-E1 cells *via* a dose-dependent manner. The ALP activity of MC3T3-E1 cells in the CPP-Ca treatment group achieved 74, 77, 91, 93, and 106 King unite/gprot at 50,100, 150, 200, and 250 μg/mL, respectively, which were increased by 7.4%, 12%, 32%, 36%, and 55% of the blank control group, respectively. In general, CPP-Ca played an important role in promoting the ALP activity of MC3T3-E1 cells and was beneficial to the differentiation of MC3T3-E1 cells.

### Effects of CPP-Ca on mineralization of MC3T3-E1 cells

**Figure 6:**
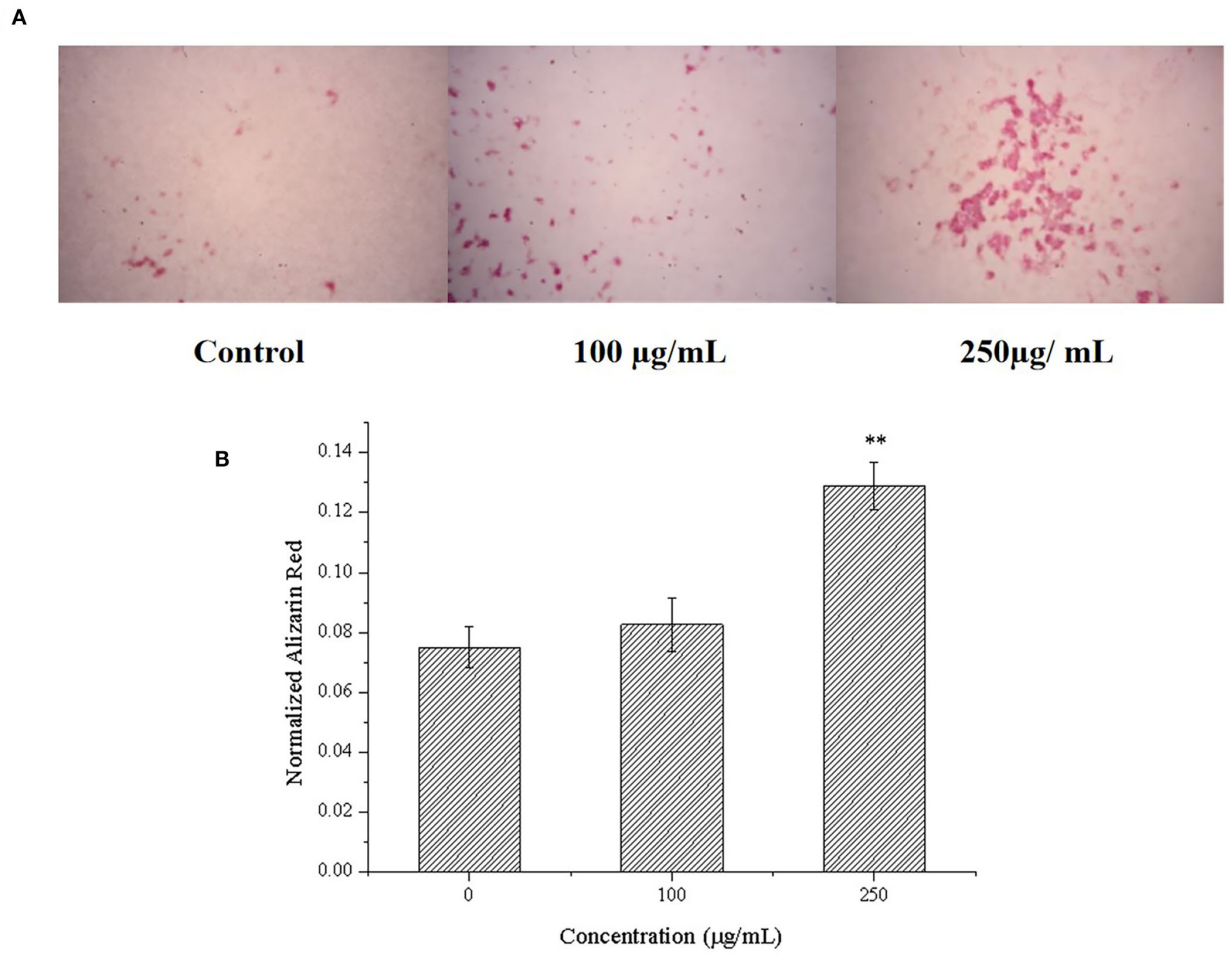
Effect of CPP-Ca on the mineralization of MC3T3-E1 cells. Alizarin red staining of mineralization nodules **(A)** and the quantification of the mineralization nodules **(B)**. *p < 0.05; **p < 0.01, compared with the control group.

Alizarin red can chelate with calcium nodules produced by the mineralization of osteoblasts to produce deep red or purple red complex, and the complex can be dissolved by cetylpyridinium chloride. Because of this, it is usually used to observe and quantitatively analyze the mineralized calcium nodules of osteoblasts (46). Therefore, we further determined the effects of CPP-Ca on the mineralization of MC3T3-E1 cells by alizarin red staining and quantitative analysis of calcium nodules with cetylpyridinium chloride (47). As shown in Figure 6, compared with the blank control group, the stained calcium nodules of the cells treated with CPP-Ca were darker and more numerous. Moreover, the calcium nodules quantitative analysis can be intuitively known that CPP-Ca significantly improved the mineralization of MC3T3-E1 cells *via* a dose-dependent manner. Compared with the blank control group, the calcium nodules were increased by 11% and 72% at 100 and 250 μg/mL, respectively. From the results of mineralization, it can be concluded that the CPP-Ca had significant effects on the mineralization of MC3T3-E1 cells.

### RNA-sequencing analysis

How to regulate osteoblast differentiation is important for the prevention and treatment of osteoporosis (48). Based on the study of osteogenic activity, RNA-seq technology was used to further reveal the mechanism of CPP-Ca promoting differentiation.

#### Analysis of DEGs

**Figure 7:**
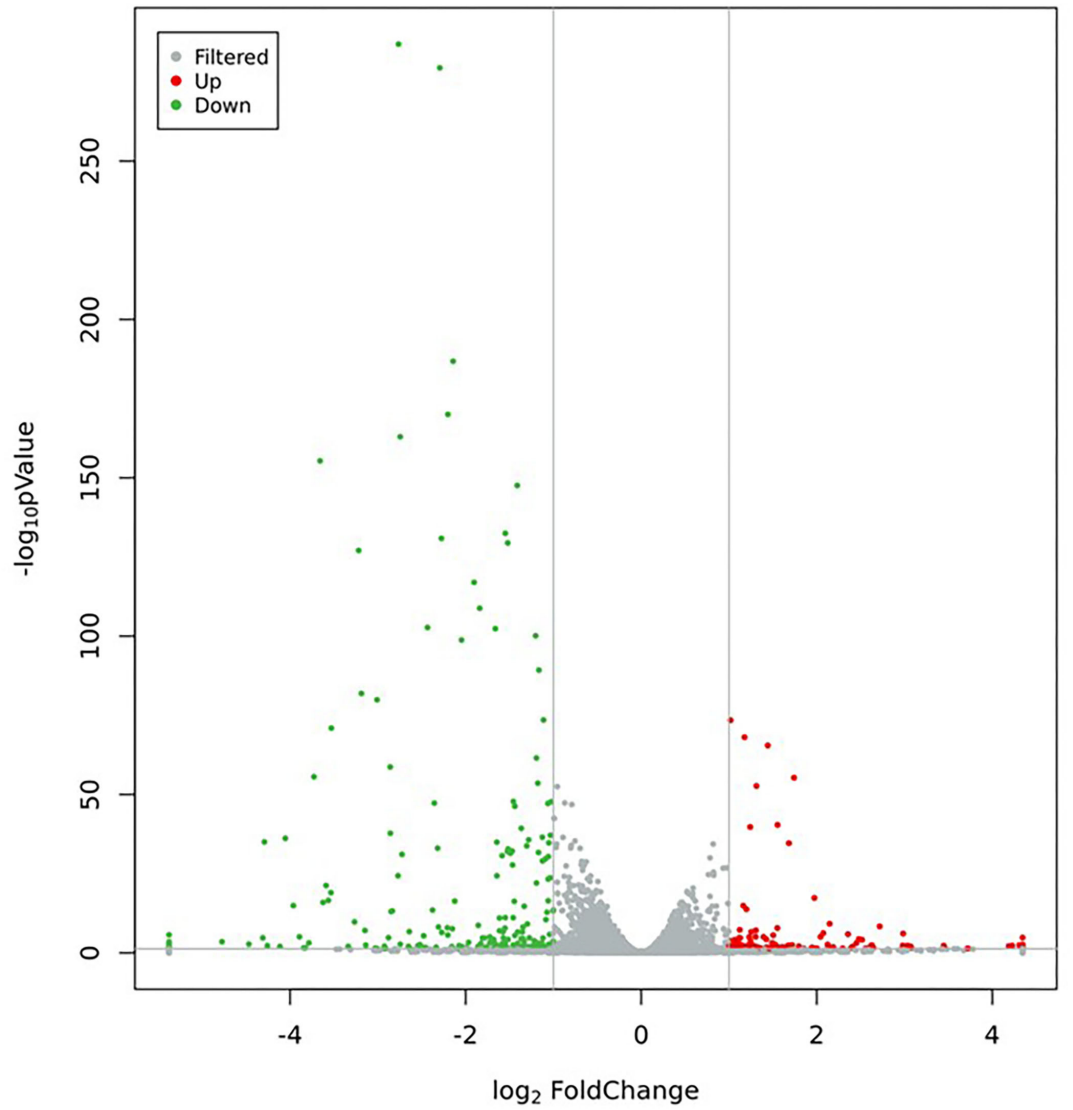
Volcano plot of DEGs (Green dots represent downregulated genes, red dots represent upregulated genes, and gray dots represent no significant difference in genes).

In this study, 321 DEGs in MC3T3-E1 cells treated with CPP-Ca were screened out (*p* < 0.05 and |log2(Fold Change)| ≥1), including 121 upregulated genes, and 200 downregulated genes (The top 15 upregulated and downregulated DEGs are shown in Tables 3, 4, respectively). Moreover, the volcano plot was used to presented the expression patterns (Figure 7), in which the red dots represented upregulated genes, and the green dots represented downregulated genes.

**Table 3:** List of the top 15 upregulated DEGs.

**Gene** **name**	**Fold** **change**	**log**_2_ **fold** **change**	***p*** **value**
Retnla	20.324	4.345	1.55E-05
Adamts16	19.818	4.308	0.003
Mmp24	18.729	4.227	0.005
Nat8f3	18.229	4.188	0.007
Ric3	13.198	3.722	0.038
Art3	13.184	3.720	0.038
Ctsc	10.911	3.447	0.005
Dner	8.424	3.074	0.028
Chl1	8.420	3.073	0.025
Myl1	8.406	3.071	0.010
Mobp	8.198	3.035	0.005
Nnat	7.915	2.984	8.24E-07
Myh4	7.890	2.980	0.004
Kcnj15	6.574	2.716	4.20E-09
Ephb1	6.189	2.629	0.003

**Table 4:** List of the top 15 downregulated DEGs.

**Gene** **name**	**Fold** **change**	**log**_2_ **fold** **change**	***p*** **value**
Vipr1	0.024	−5.379	2.04E-06
Mfsd7c	0.036	−4.775	0.0002
Smim6	0.045	−4.469	0.0017
Smagp	0.050	−4.310	1.83E-05
B4galnt3	0.051	−4.292	9.33E-36
Col13α1	0.052	−4.258	0.005
Nrros	0.057	−4.115	0.009
Adamts18	0.060	−4.054	6.78E-37
Smim5	0.064	−3.963	1.20E-15
Ptprr	0.067	−3.893	8.62E-06
Psd4	0.069	−3.843	0.025
Ptpro	0.070	−3.826	0.025
Clic3	0.072	−3.785	0.0007
Col22α1	0.075	−3.728	2.51E-56
Ncf1	0.079	−3.658	4.61E-156

#### GO enrichment analysis of DEGs

**Figure 8:**
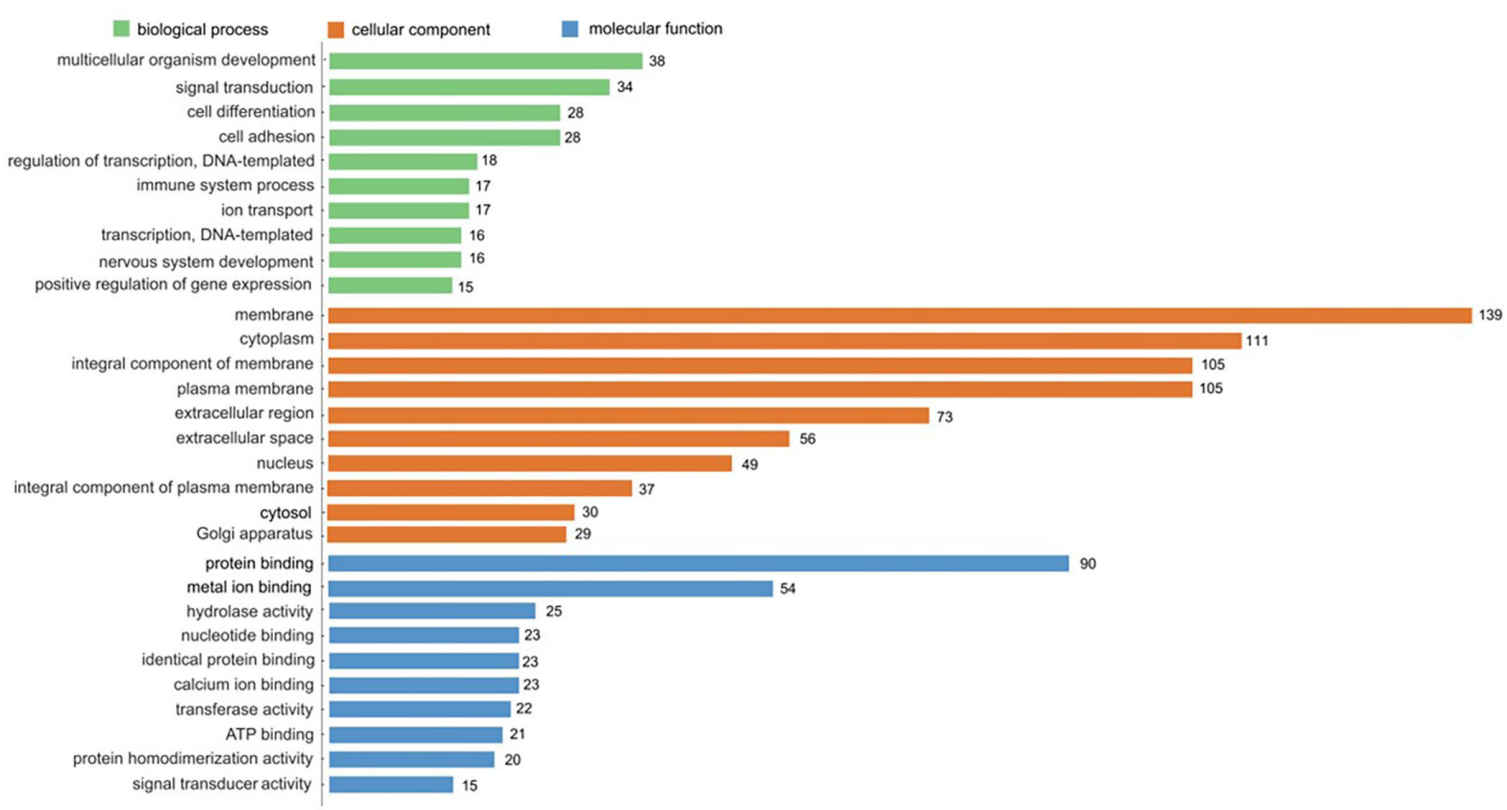
GO enrichment analysis of DEGs. The top 10 enriched in cellular component, molecular function, and biological process, respectively.

GO enrichment analysis is a comprehensive description of the gene and gene product properties in the organism. It contains three aspects: molecular function, cellular component, and biological process (49). According to the functional enrichment analysis of DEGs, the top 10 terms of GO categories and the number of genes are shown in Figure 8. The DEGs mainly participated in biological processes and were assigned to multicellular organism development (38 genes), signal transduction (34 genes), cell differentiation (28 genes), and cell adhesion (28 genes). Cellular component classification showed that most of the DEGs were located in the regions of the membrane (139 genes), cytoplasm (111 genes), integral component of membrane (105 genes), and plasma membrane (105 genes). Molecular function classification showed that the dominant functions of these DEGs were involved in protein binding (90 genes), metal ion binding (54 genes), hydrolase activity (25 genes), and nucleotide binding (23 genes). Through the comparison of biological process, cellular component, and molecular function, the DEGs accounted for a large proportion of cellular component, thus it can be inferred that DEGs mainly played an important role in the regulation of cellular components.

#### KEGG pathway analysis of DEGs

**Figure 9:**
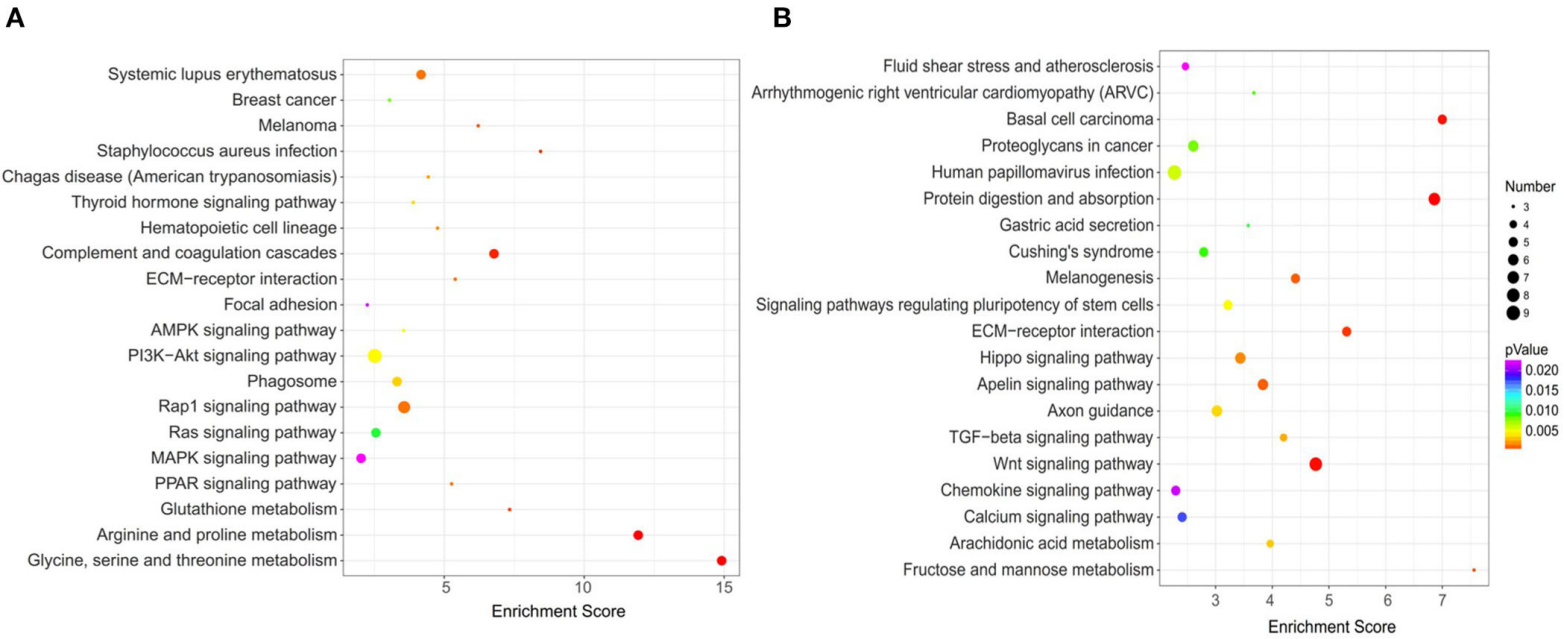
KEGG pathway enrichment analysis of DEGs. **(A)** Top 20 of enriched pathways for 121 upregulated DEGs; **(B)** Top 20 of enriched pathways for 200 downregulated DEGs.

With the advantage of the powerful graphical function, the KEGG pathway analysis can provide more intuitive and comprehensive information (19). To discern the functions of the DEGs, they were mapped using the KEGG database. The top 20 significantly enriched pathways in upregulated and downregulated DEGs are shown in Figures 9A,B, respectively. Among the significantly upregulated pathways, the significant enrichment pathways included: glycine, serine, and threonine metabolism (4 genes), MAPK signaling pathway (4 genes), complement and coagulation cascades (4 genes), PI3K-Akt signaling pathway (6 genes), AMPK signaling pathway (3 genes), and so on. While pathways that were significantly downregulated and enriched included protein digestion and absorption (7 genes), Wnt signaling pathway (8 genes), Basal cell carcinoma (5 genes), ECM-receptor interaction (5 genes), Fructose and mannose metabolism (3 genes), and so on.

PI3K-Akt signaling pathway affects bone formation and bone cells survival, thereby controlling the balance of bone density (50). Wnt signaling is a common signal transduction pathway involved in the control of a variety of biological phenomena, and osteoblast differentiation has been confirmed to be regulated by the Wnt pathway (51, 52). MAPK pathway is related to osteogenic activity, including the ERK pathway, P38 pathway, and JNK pathway (53). Specifically, the ERK pathway affects osteoblast proliferation and ALP activity, and promotes the expression of ERα, P-ERK, and Osterix. The P38 pathway can promote osteoblast mineralization and the expression of ALP and BMP. The JNK pathway is related to promote the expression of osteocalcin (OCN) and osteopontin (OPN) (54, 55). AMPK pathway regulates the cell ability and metabolic balance by sensing energy changes, thus affecting cell biological functions such as cell proliferation, apoptosis, and differentiation (56). Activation of the AMPK pathway can increase the proliferation and promote the differentiation and mineralization of MC3T3-E1 cells (57, 58). In general, CPP-Ca promoted the differentiation of MC3T3-E1 cells may be *via* these signal pathways.

It was found that IGF1 appeared in the AMPK, PI3K-Akt, and MAPK signaling pathways and may play a key role in osteoblasts differentiation. In general, CPP-Ca regulates MC3T3-E1 cell differentiation through these signaling pathways.

### Analysis of validation of DEGs using qRT-PCR

According to GO enrichment and KEGG pathway analysis, as well as considering the gene expression, six DEGs were selected for further verification by qRT-PCR, including three downregulated genes (NOTUM, WIF1, and LRP4) and three upregulated genes (APOD, OGN, and IGF1).

NOTUM is a lipase, an inhibitor of the Wnt/β-catenin signaling pathway, that inactivates Wnt by cleaving to the palmitoleate moiety phosphoric acid required for binding to the Frizzled receptor (59, 60). Brommage et al. found that the ALP activity and mineralization of osteoblasts were greatly enhanced in the absence of NOTUM, so it was concluded that the removal of NOTUM was conducive to the differentiation of osteoblasts (59). LRP4 is a member of the low-density lipoprotein family receptor (61), and it has a negative regulatory effect on the Wnt/β-catenin signaling pathway by competitively binding LRP5/LRP6 with the Wnt/FZ complex (62, 63). Loss of LRP4 may weaken the inhibition of sclerosis protein on the Wnt/β-catenin signaling pathway, which is beneficial to the differentiation of osteoblasts (64). The expression of WIF1 is closely related to osteoblast differentiation (65). WIF1 directly binds to Wnt ligands and prevents Wnt from binding to frizzled and LRP5/LRP6, thereby inhibiting the Wnt/β-catenin signaling pathway (66). Liang et al. found that Gossypol inhibited WIF1 expression and promoted osteoblast differentiation in the Wnt/β-catenin signaling pathway (67). IGF1 is a key protein in bone formation and plays an important role in the regulation of bone conversion and osteogenic growth (68–70). Yuan et al. found that IGF1 promoted the differentiation of osteoblasts (71). Furthermore, Xue et al. found that IGF1 promoted osteogenic differentiation of rat bone marrow mesenchymal stem cells by increasing TAZ expression (72). OGN, also known as an osteoinductive factor, is a member of the small leucine-rich proteoglycans family (73), secreted by myoblasts and stimulates osteoblast differentiation, and plays an important role in muscle and bone interaction (74). Chen et al. found that the overexpression of OGN was beneficial to the differentiation of osteoblasts, and thus it is beneficial to the treatment of osteoporosis (75). APOD, a 29-kDa glycoprotein (76), plays a key role in promoting osteoblast differentiation and preventing osteoporosis. Yu et al. found that APOD alleviates glucocorticoid-induced osteogenesis suppression in bone marrow mesenchymal stem cells via the PI3K/Akt pathway (77). Martineau et al. reported that APOD deficiency is associated with high-bone turnover, low bone mass, and impaired osteoblastic function in aged female mice (78). Ishii et al. found that APOD gene expression was inducive to osteogenic differentiation (79).

**Figure 10:**
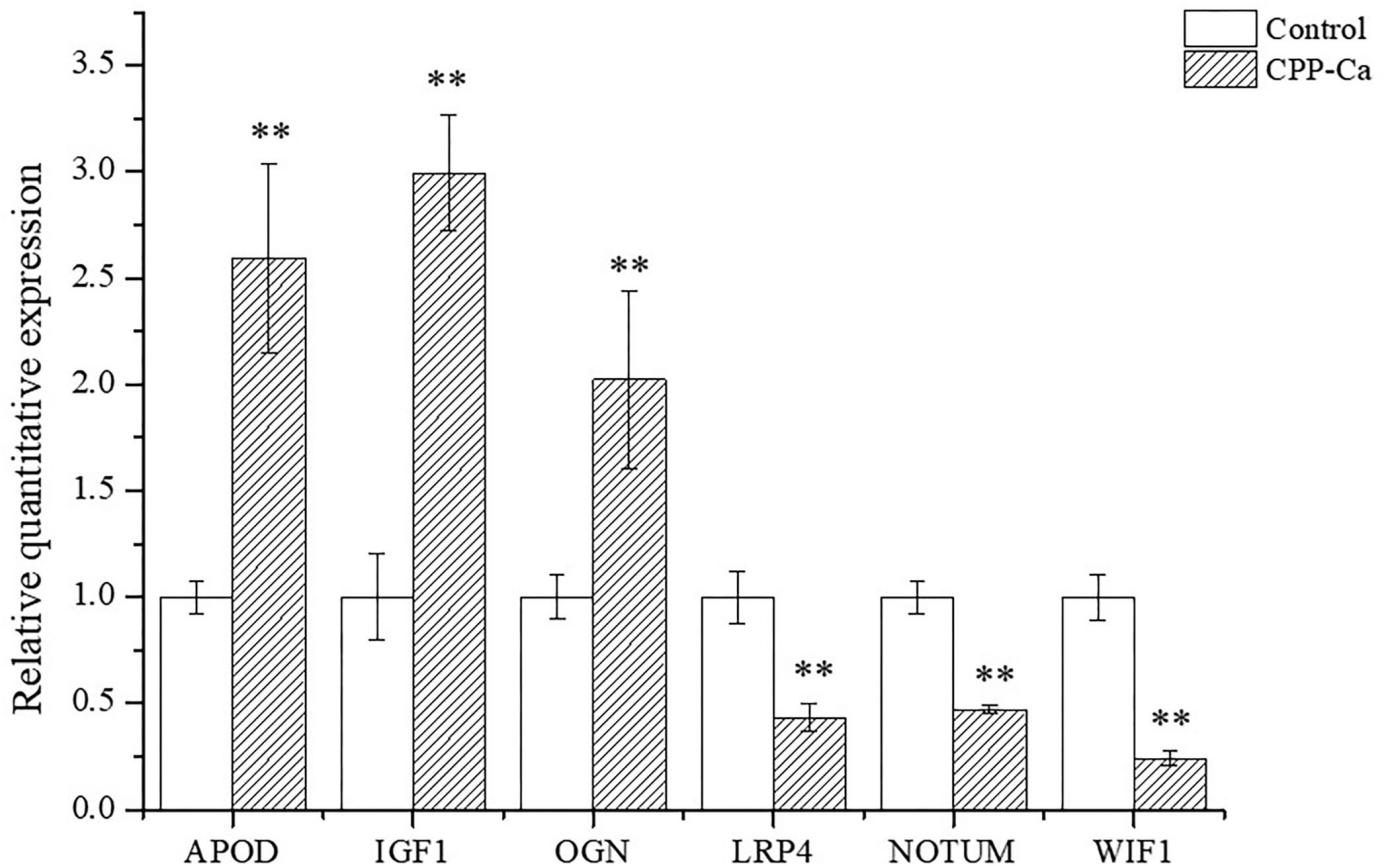
Verification of the expression profiling of DEGs by qRT-PCR. *p < 0.05; **p < 0.01, compared with the control group.

The mRNA expression levels of six DEGs are shown in Figure 10. The results of mRNA expression levels were consistent with RNA-seq analysis. Specifically, compared with the blank control, the mRNA expression levels of APOD, OGN, and IGF1in MC3T3-E1 cells treated with CPP-Ca were significantly increased 2.6, 2.0, and 3.0 times, respectively, while the mRNA expression levels of NOTUM, WIF1, and LRP4 notably decreased to 2.3, 2.1, and 4.2 times, respectively. Thus, the results of validation of DEGs suggested that CPP-Ca can promote differentiation through upregulating the expression of APOD, OGN, and IGF1 and downregulating the expression of NOTUM, WIF1, and LRP4. Therefore, NOTUM, WIF1, LRP4, APOD, OGN, and IGF1 played important roles in the differentiation of MC3T3-E1 cells treated with CPP-Ca.

## Conclusion

In this study, the preparation of CPP-Ca was optimized and proved that CPP-Ca has significant activity on the proliferation, differentiation, and mineralization of MC3T3-E1 cells. There were 321 DEGs in MC3T3-E1 cells treated with CPP-Ca, which mainly played an important role in regulating cellular component. The differentiation mechanism of CPP-Ca on MC3T3-E1 cells may be related to the regulation of the AMPK signaling pathway, PI3K-Akt signaling pathway, MAPK signaling pathway, and Wnt signaling pathway, and the expression of NOTUM, WIF1, LRP4, APOD, OGN, and IGF1. This study provides a theoretical basis for the application of CPP-Ca as a food additive in the prevention and treatment of osteoporosis.

## Data availability statement

The data presented in the study are deposited in the Sequence Read Archive (SRA) of NCBI repository, accession to cite for these SRA data: PRJNA849366, submission ID: SUB11761368 (https://www.ncbi.nlm.nih.gov/sra/PRJNA849366).

## Author contributions

JM, YC, SX, and SD provided the experimental design, project administration, and funding acquisition. WL and ZL performed the initial literature research. WH, LL, and YD carried out experimental research, analyzed the data, and wrote the manuscript. All authors contributed to the article and approved the submitted version.

## Funding

This work was supported by the Natural Science Foundation of Guangdong Province (2020A1515010371), the Open Project of State Key Laboratory of Natural Medicines (No. SKLNMKF202008), the State Key Laboratory for Chemistry and Molecular Engineering of Medicinal Resources (Guangxi Normal University) (CMEMR2020-B07), and the Solid-state Fermentation Resource Utilization Key Laboratory of Sichuan Province (No. 2019GTY003).

## Conflict of interest

The authors declare that the research was conducted in the absence of any commercial or financial relationships that could be construed as a potential conflict of interest.

## Publisher's note

All claims expressed in this article are solely those of the authors and do not necessarily represent those of their affiliated organizations, or those of the publisher, the editors and the reviewers. Any product that may be evaluated in this article, or claim that may be made by its manufacturer, is not guaranteed or endorsed by the publisher.
